# Misfit-Strain Phase Diagram, Electromechanical and Electrocaloric Responses in Epitaxial PIN–PMN–PT Thin Films

**DOI:** 10.3390/ma15217660

**Published:** 2022-10-31

**Authors:** Yun Ou, Yingying Wu, Jinlin Peng

**Affiliations:** 1 Hunan Provincial Key Laboratory of Health Maintenance for Mechanical Equipment, Hunan University of Science and Technology, Xiangtan 411201, China; 2 School of Materials Science and Engineering, Hunan University of Science and Technology, Xiangtan 411201, China; 3 All-Solid-State Energy Storage Materials and Devices Key Laboratory of Hunan Province, College of Information and Electronic Engineering, Hunan City University, Yiyang 413002, China

**Keywords:** misfit strain, PIN–PMN–PT, electrocaloric effect, ferroelectric thin films

## Abstract

*x*Pb(In_1/2_Nb_1/2_)O_3_-(1−*x*−*y*)Pb(Mg_1/3_Nb_2/3_)O_3_−*y*PbTiO_3_ (PIN–PMN–PT) bulks possess excellent electromechanical coupling and dielectric properties, but the corresponding epitaxial PIN–PMN–PT thin films have not yet been explored. This paper adopts a nonlinear thermodynamics analysis to investigate the influences of misfit strains on the phase structures, electromechanical properties, and electrocaloric responses in epitaxial PIN–PMN–PT thin films. The misfit strain–temperature phase diagram was constructed. The results reveal that the PIN–PMN–PT thin films may exist in tetragonal *c*-, orthorhombic *aa*-, monoclinic *M*-, and paraelectric PE phases. It is also found that the *c*-*M* and *aa*-PE phase boundaries exhibit a superior dielectric constant $\varepsilon_{11}$ which reached 1.979 × 10^6^ with u_m_ = −0.494%, as well as the *c*-*M* phase boundary showing a large piezoelectric response *d*_15_ which reached 1.64 × 10^5^ pm/V. In comparison, the c-PE and M-aa phase boundaries exhibit a superior dielectric constant ε_33_ over 1 × 10^5^ around um = 0.316% and the piezoelectric response *d*_33_ reached 7235 pm/V. The large electrocaloric responses appear near the paraelectric- ferroelectric phase boundary. These insights offer a guidance for experiments in epitaxial PIN–PMN–PT thin films.

## 1. Introduction

Ferroelectric materials, which exhibit a polarization with electromechanical coupling [1,2], have been employed in actuators, sensors, piezoelectric energy harvesters, storage devices, etc. [3,4]. Excellent performance is the key to the application of ferroelectric materials, which prompts people to continuously explore ferroelectric materials with an excellent performance [5,6,7]. Piezoelectric materials contain defects such as ferroelectric domains, oxygen vacancies, defect dipoles, and the strain [8,9,10]. PbMg_1/3_Nb_2/3_O_3_−PbTiO_3_(PMN–PT) can reach an ultrahigh piezoelectric response (d_33_ > 2000 pC/N) and has electromechanical coupling factors (k_33_ > 0.9) [11], which have attracted much attention [12,13,14,15]. The novel ternary compound *x*Pb(In_1/2_Nb_1/2_)O_3_-(1−*x*−*y*)Pb(Mg_1/3_Nb_2/3_)O_3_−*y*PbTiO_3_ (PIN–PMN–PT) has been proposed to increase the coercive field and phase transition temperature of these materials without a change in the piezoelectric properties [16,17,18]. Thus, compared with PMN–PT, PIN–PMN–PT remains a more ferroelectric state which is stable under high temperatures.

There are more studies on PIN–PMN–PT bulk. For instance, in the experimental aspect, Li et al. [11] investigated the ferroelectric, dielectric, elastic, piezoelectric, and electromechanical properties of tetragonal PIN–PMN–PT crystals. The electromechanical coupling exhibited a high dc bias electric field stability compared to its rhombohedral counterpart, and the single domain piezoelectric coefficients d_33_ and d_15_ were found to be 530 and 2350 pC/N, respectively. Lin et al. [18] studied the piezoelectric thermal stability of PIN–PMN–PT ternary ceramics near the morphotropic phase boundary. The resulting temperature-dependent piezoelectric effects in the PIN–PMN–PT ceramics indicate that this ternary ceramics system within the MPB region shows a better temperature stability and increased usable temperature range compared with the PMN–32PT single crystals. In the theoretical aspect, Lv et al. developed a Landau–Devonshire energy functional for PIN–PMN-PT to investigate the phase transformation, phase diagrams, and the electromechanical properties of the PIN–PMN–PT single crystal [19], which match well with the experiments. On the contrary, research on PIN–PMN–PT thin films has been rare. Compared to bulk films, thin films are grown on a substrate, which impose a strain constraint due to a substrate lattice mismatch [9,20,21,22,23]. It is known that the misfit strain can, in general, influence the phase structures and electromechanical properties in thin films [24]. The films have wider applications and a better adjustment than the bulks [8]. However, the influences of the misfit strain on the phase structures, electromechanical properties, and electrocaloric response in the epitaxial PIN–PMN–PT thin films have been lacking, which hinders the corresponding experimental studies. Notice that the misfit strain in the films is caused by the mismatch of lattice between the substrate and the film, which can be relaxed by a defect [25,26,27,28,29], such as a dislocation in the thicker films [30]. Thus, this article employs a nonlinear thermodynamic analysis to establish the misfit strain–temperature phase diagram for PIN–PMN–PT (26PIN–42PMN–32PT) thin films, which the stoichiometric composition are described by atomic%, from which the influences of the misfit strain on the phase structures, electromechanical properties, and electrocaloric responses are studied, offering a guidance for PIN–PMN–PT thin film experiments.

The structure of this article is as follows: the theory of nonlinear thermodynamics for ferroelectric thin films is outlined in Section 2, including the calculation methods for the electromechanical properties and electrocaloric response. The influences of the misfit strain on the phase structures, electromechanical properties, and electrocaloric responses of PIN–PMN–PT thin films are investigated in Section 3. The important discoveries and conclusions are summarized in Section 4.

## 2. Computational Model

### 2.1. Thermodynamic Potential of Thin Films and Electromechanical Properties

Lv et al. [19] developed a tenth-order Landau–Devonshire energy function for PIN–PMN–PT, which was restricted to the bulk structures. We consider the epitaxial PMN–PT–PIN thin films subjected to the in-plane biaxial misfit strain $u_{m}$, leading to the mixed boundary conditions as below [30,31]:

$$\varepsilon_{1}=\varepsilon_{2}=u_{m},\;\varepsilon_{6}=0,\;\sigma_{3}=\sigma_{4}=\sigma_{5}=0$$

Thus, the thermodynamic potential of PMN–PT–PIN thin films can be obtained from the standard Gibbs function using the Legendre transform [30,31]:

(1) $$\begin{array}{cc} G= & \alpha_{1}{}^{*}(P_{1}{}^{2}+P_{2}{}^{2})+\alpha_{11}{}^{*}(P_{1}{}^{4}+P_{2}{}^{4})+\alpha_{12}^{*}{\left(P_{1}P_{2}\right)}^{2}+\alpha_{13}^{*}P_{3}{}^{2}(P_{1}{}^{2}+P_{2}{}^{2})+\alpha_{3}^{*}P_{3}{}^{2}+\alpha_{33}^{*}P_{3}{}^{4}+\alpha_{123}{\left(P_{1}P_{2}P_{3}\right)}^{2}\;\\ \; & +\alpha_{111}(P_{6}{}^{2}+P_{6}{}^{2}+P_{6}{}^{3})+\alpha_{1111}(P_{1}^{8}+P_{2}^{8}+P_{3}^{8})+\alpha_{1112}[P_{3}^{2}(P_{1}^{6}+P_{2}^{6})+P_{2}^{2}(P_{1}^{6}+P_{3}^{6})+P_{1}^{2}(P_{3}^{6}+P_{2}^{6})]\\ \; & +\alpha_{112}[P_{3}^{2}(P_{1}^{4}+P_{2}^{4})+P_{2}^{2}(P_{1}^{4}+P_{3}^{4})+P_{1}^{2}(P_{3}^{4}+P_{2}^{4})]\\ \; & +\alpha_{1122}[{\left(P_{1}P_{2}\right)}^{4}+{\left(P_{3}P_{2}\right)}^{4}+{\left(P_{1}P_{3}\right)}^{4}]+\alpha_{1123}[{\left(P_{1}^{2}P_{2}P_{3}\right)}^{2}+{\left(P_{2}^{2}P_{1}P_{3}\right)}^{2}+{\left(P_{3}^{2}P_{1}P_{2}\right)}^{2}]\\ \; & +\alpha_{11111}(P_{1}^{10}+P_{2}^{10}+P_{3}^{10})+\alpha_{11122}[P_{3}^{8}(P_{1}^{2}+P_{2}^{2})+P_{2}^{8}(P_{1}^{2}+P_{3}^{2})+P_{1}^{8}(P_{3}^{2}+P_{2}^{2})]\\ \; & +\alpha_{11122}[P_{3}^{6}(P_{1}^{4}+P_{2}^{4})+P_{2}^{6}(P_{1}^{4}+P_{3}^{4})+P_{1}^{6}(P_{3}^{4}+P_{2}^{4})]+\alpha_{11223}[{\left(P_{1}^{2}P_{2}^{2}P_{3}\right)}^{2}+{\left(P_{1}^{2}P_{3}^{2}P_{2}\right)}^{2}+{\left(P_{2}^{2}P_{3}^{2}P_{1}\right)}^{2}]\\ \; & +\alpha_{11123}[{\left(P_{1}P_{2}P_{3}^{3}\right)}^{2}+{\left(P_{1}P_{3}P_{2}^{3}\right)}^{2}+{\left(P_{3}P_{2}P_{1}^{3}\right)}^{2}]+\frac{u_{m}{}^{2}}{S_{11}+S_{12}}-E_{1}P_{1}-E_{2}P_{2}-E_{3}P_{3} \end{array}$$

With
$\alpha_{1}^{*}=\alpha_{1}-\frac{u_{m}(Q_{11}+Q_{12})}{S_{11}+S_{12}},$                                                                                                            (2)$\alpha_{3}^{*}=\alpha_{1}-\frac{2Q_{12}u_{m}}{S_{11}+S_{12}},$(3)$\alpha_{11}^{*}=\alpha_{11}+\frac{S_{11}(Q_{11}{}^{2}+Q_{12}{}^{2})-2Q_{11}Q_{12}S_{12}}{2(S_{11}{}^{2}-S_{12}{}^{2})},$(4)$\alpha_{33}^{*}=\alpha_{11}+\frac{Q_{12}{}^{2}}{S_{11}+S_{12}},$(5)$\alpha_{12}^{*}=\alpha_{12}-\frac{S_{12}(Q_{11}{}^{2}+Q_{12}{}^{2})-2Q_{11}Q_{12}S_{11}}{2(S_{11}{}^{2}-S_{12}{}^{2})}+\frac{Q_{44}{}^{2}}{2S_{44}},$(6)$\alpha_{13}^{*}=\alpha_{12}+\frac{Q_{12}(Q_{11}+Q_{12})}{S_{11}+S_{12}},\;$(7)
where $P_{i}$ and $E_{i}$ represent the components of polarization and external electric fields; $\alpha_{1}$, $\alpha_{ij}$, $\alpha_{ijk}$, $\alpha_{ijkl}$, and $\alpha_{ijklm}$ are the dielectric constants under constant stress; $\alpha_{1}^{*}$ and $\alpha_{ij}^{*}$ are the normalized dielectric constants; $S_{ij}$ represents the elastic compliance coefficient; and $Q_{ij}$ represents the electrostrictive coefficient. The material parameters used in the calculations are listed in Table 1.

According to the principle of the minimum energy, the polarization components of the thin films at stable configurations can be computed by solving the system of the equation:

$$\frac{\partial G}{\partial P_{1}}=0,\;\frac{\partial G}{\partial P_{2}}=0,\;\frac{\partial G}{\partial P_{3}}=0$$

With the computed polarization components ($P_{1},\;P_{2},\;P_{3}$), the relative dielectric constant of the thin film can be calculated [32]:

(8) $$\varepsilon_{ij}=1+\frac{\eta_{ij}}{\varepsilon_{0}}$$

where

**Table 1:** The material parameters used in the calculations [11,19,33].

Coefficients	Values	Units
$\alpha_{1}$	$3.816\times 10^{4}\left(T-182\right)$	$C^{-2}m^{2}N,\;\mathrm{and}\;T\;\mathrm{in}\;{}^{\circ}C$
$\alpha_{11}$	$-1.212\times 10^{7}$	$C^{-4}m^{6}N$
$\alpha_{12}$	$-1.285\times 10^{7}$	$C^{-4}m^{6}N$
$\alpha_{111}$	$9.424\times 10^{7}$	$C^{-6}m^{10}N$
$\alpha_{112}$	$1.550\times 10^{8}$	$C^{-6}m^{10}N$
$\alpha_{123}$	$4.716\times 10^{9}$	$C^{-6}m^{10}N$
$\alpha_{1111}$	$3.190\times 10^{7}$	$C^{-8}m^{14}N$
$\alpha_{1112}$	$2.521\times 10^{9}$	$C^{-8}m^{14}N$
$\alpha_{1122}$	$-1.993\times 10^{9}$	$C^{-8}m^{14}N$
$\alpha_{1123}$	$-3.956\times 10^{10}$	$C^{-8}m^{14}N$
$\alpha_{11112}$	$-8.865\times 10^{9}$	$C^{-10}m^{18}N$
$\alpha_{11223}$	$1.717\times 10^{11}$	$C^{-10}m^{18}N$
$\alpha_{11123}$	$8.946\times 10^{10}$	$C^{-10}m^{18}N$
$\alpha_{11111}$	0	$C^{-10}m^{18}N$
$\alpha_{11122}$	0	$C^{-10}m^{18}N$
$Q_{11}$	0.066	$m^{4}/C^{2}$
$Q_{12}$	−0.032	$m^{4}/C^{2}$
$Q_{44}$	0.023	$m^{4}/C^{2}$
$S_{11}$	$12.3\times 10^{-12}$	$m^{2}/N$
$S_{12}$	$-7.1\times 10^{-12}$	$m^{2}/N$
$S_{44}$	$15.1\times 10^{-12}$	$m^{2}/N$
$C_{latt}$	$2.697\times 10^{6}$	$J/m^{3}K$

(9) $$\eta =\chi^{-1}=\left[\begin{array}{ccc} \frac{\partial^{2}G}{\partial P_{1}\partial P_{1}} & \frac{\partial^{2}G}{\partial P_{1}\partial P_{2}} & \frac{\partial^{2}G}{\partial P_{1}\partial P_{3}}\\ \frac{\partial^{2}G}{\partial P_{2}\partial P_{1}} & \frac{\partial^{2}G}{\partial P_{2}\partial P_{2}} & \frac{\partial^{2}G}{\partial P_{2}\partial P_{3}}\\ \frac{\partial^{2}G}{\partial P_{3}\partial P_{1}} & \frac{\partial^{2}G}{\partial P_{3}\partial P_{2}} & \frac{\partial^{2}G}{\partial P_{3}\partial P_{3}} \end{array}\right]$$

For (001)-oriented thin films, the piezoelectric coefficients $d_{in}$ can be computed [22]:

(10) $$d_{in}=\frac{\partial s_{n}}{\partial P_{1}}\eta_{i1}+\frac{\partial s_{n}}{\partial P_{2}}\eta_{i2}+\frac{\partial s_{n}}{\partial P_{3}}\eta_{i3},$$

where the strain $S_{i}$ is obtained from the stress–strain relation [30]. The main focus has been placed on the significant piezoelectric coefficients $d_{15}$ and $d_{33}$. The strain components used for calculating the piezoelectric coefficients $d_{15}$ and $d_{33}$ are given as [32]:

(11) $$\begin{array}{cc} s_{3}= & \frac{2u_{m}S_{12}}{S_{11}+S_{12}}+\left[Q_{12}-\frac{S_{12}(Q_{11}+Q_{12})}{S_{11}+S_{12}}(P_{1}{}^{2}+P_{2}{}^{2})+\left(Q_{11}-\frac{2S_{12}Q_{12}}{S_{11}+S_{12}}\right)P_{3}{}^{2}\right]\\ s_{5}= & Q_{44}P_{1}P_{3}\;\;\;\;\;\;\;\;\;\;\;\;\;\;\;\;\; \end{array}$$

### 2.2. Adiabatic Temperature Change in Electrocaloric Response

The electrocaloric (EC) effect is a phenomenon in a dielectric material that shows an adiabatic temperature change ΔT under an applied electric field change, or the entropy change induced by the isothermal conditions [34]. Following the method developed by Liu et al. in the previous work [35,36], we use an entropy-based analysis to calculate the EC adiabatic temperature change ΔT for the epitaxial PMN–PT–PIN thin films. In the literature [35,36,37], the total entropy $S_{total}$ of a ferroelectric thin film can be written as the sum of the dipolar entropy $S_{dip}$ and the lattice entropy $S_{latt}$,

(12) $$S_{total}\left(E,T\right)=S_{dip}\left(E,T\right)+S_{latt}\left(T\right)$$

In Equation (12), $S_{dip}\left(E,T\right)$ is the contribution from the dipolar degree of freedom, which is a function of polarization, depending on the working temperature *T*, the external electric field E, and the misfit strain. $S_{latt}\left(T\right)$ is assumed to be only correlated to the lattice contribution. Under the adiabatic condition, the total entropy change of the thin film is zero, leading to

(13) $$S_{latt}\left(T_{f}\right)-S_{latt}\left(T_{i}\right)=-[S_{dip}\left(E_{f},T_{f}\right)-S_{dip}\left(E_{i},T_{i}\right)]$$

where subscripts *i* and *f* correspond to the initial and final states, respectively. The change in y $S_{latt}$ can be approximated by

(14) $$S_{latt}\left(T_{f}\right)-S_{latt}\left(T_{i}\right)=\int_{T_{i}}^{T_{f}}\frac{C_{latt}\left(T\right)}{T}dT\approx C_{latt}\left(T_{i}\right)\ln\left(\frac{T_{f}}{T_{i}}\right)$$

Note that $C_{latt}$ is the lattice heat capacity per unit volume. Combining Equations (13) and (14), the final state temperature $T_{f}$ can be calculated by

(15) $$T_{f}=T_{i}exp\left\{-\frac{1}{C_{latt}}\left[S_{dip}\left(E_{f},T_{f}\right)-S_{dip}\left(E_{i},T_{i}\right)\right]\right\}$$

Thus, the adiabatic temperature change in the electrocaloric response is given by

(16) $$\Delta T=T_{f}-T_{i}=T_{i}exp\left\{-\frac{1}{C_{latt}}\left[S_{dip}\left(E_{f},T_{f}\right)-S_{dip}\left(E_{i},T_{i}\right)\right]\right\}-T_{i}$$

where the dipolar entropy $S_{dip}$ is associated with the dipolar free energy ofthe ferroelectric thin films and can be determined by [35,36,37].

(17) $$S_{dip}\left(E,T\right)=-{\left(\frac{\partial G}{\partial T}\right)}_{E}$$

## 3. Results and Discussion

The above nonlinear thermodynamics method is adopted to analyze the influences of the misfit strain on the phase structures, electromechanical properties, and electrocaloric response in PIN–PMN–PT thin films. The material parameters used in the calculations are listed in Table 1 [11,19,33], which accurately reproduce the phase diagrams and electromechanical properties in the PIN–PMN–PT bulk, indicating the reliability of the material parameters.

**Figure 1:**
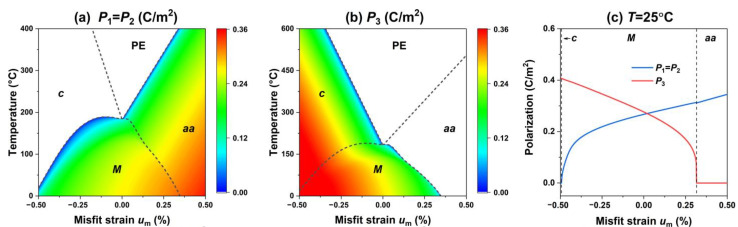
(**a**,**b**) The misfit strain-temperature phase diagrams in the absence of external electric field, and the corresponding (**a**) in-plane polarization components *P*_1_, *P*_2_, and (**b**) the out-of-plane polarization components *P*_3_. (**c**) The corresponding polarization components as functions of misfit strain *u*_m_ at room temperature. The color bar illustrates the value of polarization.

We first investigate the influence of the misfit strain on the phase structures of the PIN–PMN–PT thin films, as shown in Figure 1. Figure 1a,b show that under the in-plane biaxial misfit strain, PIN–PMN–PT thin films may exhibit the four phase structures, and their polarization characteristics are summarized in Table 2. It can be seen that the tetragonal *c* phase is easily formed under a compressive strain, and the orthorhombic *aa* phase is more easily formed under a tensile strain. The tetragonal phase c has a polarization along the [001]-direction. In contrast, the monoclinic *M* phase can exist under both a compressive strain and a tensile strain. At high temperatures, the paraelectric PE phase is formed. To more clearly observe the variation of polarization with the mismatch strain more clearly, the change in the polarization components with the misfit strain at room temperature is plotted in Figure 1c. It can be seen that with the misfit strain change from compressive to tensile, the PIN–PMN–PT films exhibit a tetragonal *c* phase, monoclinic *M* phase, and orthorhombic aa phase in turn. The out-of-plane component $P_{3}$ decreases within the monoclinic *M* phase. In contrast, the in-plane component $P_{1}$ exhibits the opposite trend. At room temperature, the *c-M* phase boundary is around *u*_m_ = −0.49%, while the *M*-*aa* phase boundary is around *u*_m_ = 0.315%.

**Figure 2:**
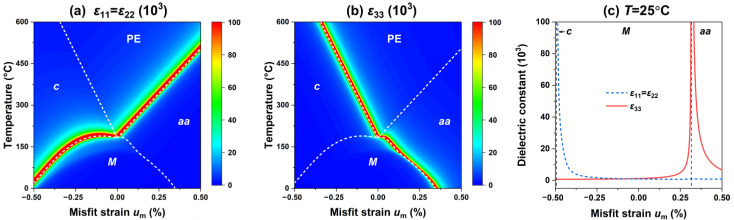
The dielectric constants (**a**) $\varepsilon_{11}$ and $\varepsilon_{22}$, and (**b**) $\varepsilon_{33}$ as a function of misfit strain and temperature. (**c**) The corresponding dielectric constants as functions of misfit strain *u*_m_ at room temperature.

**Figure 3:**
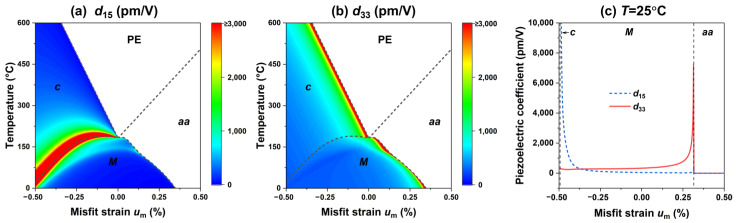
The piezoelectric coefficients (**a**) *d*_15_, and (**b**) *d*_33_ as a function of misfit strain and temperature. (**c**) The corresponding piezoelectric coefficients as function of misfit strain *u*_m_ at room temperature.

Next, we investigate the influence of the misfit strain on the electromechanical properties of the PIN–PMN–PT thin films, including the dielectric and piezoelectric responses. Figure 2 presents the dielectric constants $\varepsilon_{11}$, $\varepsilon_{22}$, and $\varepsilon_{33}$ of the PIN–PMN–PT films at different temperatures and misfit strains. Due to the in-plane biaxial misfit strain on the thin films, it is expected that $\varepsilon_{11}=\varepsilon_{22}$. Figure 2a shows the excellent transverse permittivity in the vicinity of the *c*-*M* phase boundary and the *aa*-PE phase boundary. Figure 2b shows an excellent longitudinal permittivity in the vicinity of the *c*-PE phase boundary and the *M*-*aa* phase boundary. Figure 2c shows the trend of the dielectric constant at room temperature with respect to the misfit strain. Similarly, the dielectric response enhancement at the phase boundary is also observed in the BaTiO_3_ [22] and BiFeO_3_ [24,38] thin films due to the abrupt change in the polarization slope near the phase boundary. The sudden change in the polarization slope also causes the PIN–PMN–PT film to exhibit an excellent piezoelectric response near the phase boundary, as shown in Figure 3a,b, where the *c*-*M* phase boundary has an excellent transverse piezoelectric response $d_{15}$, the *c*-PE phase boundary and *M*-*aa* phase boundary exhibit an excellent longitudinal piezoelectric response $d_{33}$. The piezoelectric response of the PIN–PMN–PT thin film at room temperature is shown in Figure 3c, which reaches a peak at the *c*-*M* phase boundary, and a peak at the *M*-*aa* phase boundary.

**Figure 4:**
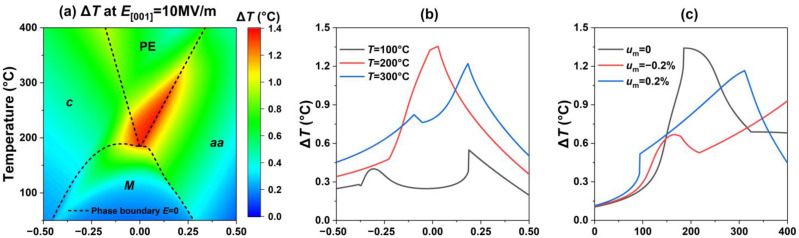
(**a**) The electrocaloric temperature change ∆*T* as a function of misfit strain and temperature under an electric field change ∆*E* = 10 MV/m. (**b**) The corresponding ∆*T* at a fixed temperature. (**c**) The corresponding ∆*T* at fixed misfit strain.

Finally, we investigate the influence of the misfit strain on the adiabatic temperature change ∆*T* in the electrocaloric response in PIN–PMN–PT thin films, as shown in Figure 4, where the electrical field is applied along the [001] direction with a variation (ΔE) of 10 MV/m. The results show that large electrocaloric responses ∆*T* appear near the ferroelectric–paraelectric phase boundary at high working temperature because the dipoles in the paraelectric PE phase are easier to reorient when the external electrical field is changed. The corresponding ∆*T* at a fixed temperature and under a fixed misfit strain are shown in Figure 4b,c, where the peaks of the EC responses ∆*T* near the phase boundaries can be observed more clearly, suggesting that an appropriate misfit strain can enhance the EC response of the PIN–PMN–PT thin films.

## 4. Conclusions

In summary, we adopt a nonlinear thermodynamics analysis to study the influences of misfit strains on the phase structures, electromechanical properties, and electrocaloric responses of epitaxial PIN–PMN–PT thin films. It is found that the PIN–PMN–PT thin films may appear in tetragonal c-, orthorhombic aa-, monoclinic M-, and paraelectric PE phases. We also found that the *c*-*M* and *aa*-PE phase boundaries show a superior dielectric constant, $\varepsilon_{11}$, as well as the *c*-*M* phase boundary having a large piezoelectric response, *d*_15_, while the *c*-PE and *M*-*aa* phase boundaries show a superior dielectric constant, $\varepsilon_{33}$, and the piezoelectric response *d*_33_. The adiabatic temperature change ∆*T* indicates that the paraelectric–ferroelectric phase boundary shows a large electrocaloric response. The findings offer guidance for PIN–PMN–PT thin film experiments.

## Figures and Tables

**Table 2:** The polarization components of the epitaxial PIN–PMN–PT thin films in the absence of an external electric field.

Phase	Polarization
Paraelectric PE	*P*_1_ = *P*_2_ = *P*_3_ = 0
Tetragonal *c*	*P*_1_ = *P*_2_ = 0, *P*_3_ ≠ 0
Orthorhombic *aa*	*P*_3_ = 0, |*P*_1_| = |*P*_2_| ≠ 0
Monoclinic *M*	|*P*_1_| = |*P*_2_| ≠ 0, *P*_3_ ≠ 0

## Data Availability

Not applicable.
